# STEM and Non-STEM Misconceptions About Evolution: Findings from 5 Years of Data

**DOI:** 10.1007/s11191-023-00428-5

**Published:** 2023-03-27

**Authors:** Pablo Antonio Archila, Silvia Restrepo, Anne‑Marie Truscott de Mejía, Jorge Molina

**Affiliations:** 1 Vice-Presidency of Research and Creation, Universidad de los Andes, Cra 1 Nº 18A-12, 111711 Bogotá, Colombia; 2 School of Education, Universidad de los Andes, Cra 1 Nº 18A-12, 111711 Bogotá, Colombia; 3 Department of Biological Sciences, Universidad de los Andes, Cra 1 Nº 18A-12, 111711 Bogotá, Colombia

## Abstract

Even though it is widely held that the theory of evolution is one of the pillars of the biological sciences, as we begin the third decade of the twenty-first century, it is alarming how little we know about science, technology, engineering, and mathematics (STEM) majors and non-STEM majors’ misconceptions about evolution in countries such as Brazil, Chile, Colombia, and Greece, to name a few. The situation is even more complicated if we acknowledge that contemporary educational approaches (e.g., student-centered learning) mean that students’ misconceptions are one of the multiple aspects that influence the construction of meaningful learning. Here, we present a picture of Colombian STEM/non-STEM majors’ misconceptions about evolution. Participants were 547 students from different STEM/non-STEM majors (278 females and 269 males, 16–24 years old). During 5 years (10 academic semesters), data were collected from students’ responses to an 11-item questionnaire administered in a Colombian university. We hypothesized that the academic semester within these 5 years in which each student completed the instrument as well as respondents’ age, gender, and/or major may influence their misconceptions about evolution. Results reveal that participants had a moderate understanding of evolution. Also, we found a limited understanding of microevolution among participants. Furthermore, cross-sectional analyses of differences in undergraduates’ responses across demographic variables showed that despite apparent differences, these were not reliable since the differences were not statistically significant. Implications for evolution education are discussed.

## Introduction

An integral understanding of the role of evolution education in the world requires that we hear and value the voices of people from as many different countries as possible, going beyond the case of the USA. This is one core reflection along the twenty-four chapters of the book *Evolution Education Around the Globe* (Deniz & Borgerding, 2018a). This reflection is legitimate and desirable once we bear in mind that “the theory of evolution is [one of] the backbone[s] of the biological sciences” (Archila & Molina, 2020, p. 1619). There are several reasons that support this assertion. Ayala (2013) provides us with at least five of them. First, genetic, morphological, and physiological similarities and differences of organisms can be explained by this theory. Second, Darwinian evolution helps us to understand the constantly evolving pathogenic organisms (e.g., some bacteria, viruses), which is particularly important for the effective development of genuine ways to protect ourselves against the diseases they cause (e.g., Coronavirus disease 2019). Third, evolutionary theory is fundamental to be able to answer the question of why there are so many different kinds of organisms on this planet. Fourth, this theory has led to improvements in multiple domains, such as agriculture and medicine. And five, knowledge of evolution has been successfully applied in many fields outside biology (e.g., chemistry and software engineering).

For all the reasons just mentioned, it makes sense to find that evolutionary theory is commonly included in science education standards, such as *Next Generation Science Standards: For States, by States* (Next Generation Science Standards Lead States, 2013). In practice, however, as we begin the third decade of the twenty-first century, we still know little about students’ misconceptions about evolution in various countries around the globe. To have an idea of how problematic this is, we should bear in mind that contemporary educational approaches mean that being informed about students’ misconceptions on any topic is fundamental in the construction of meaningful learning. Supporters of student-centered learning approaches, for example, maintain that instructors take a step in the right direction towards the construction of student-centered learning environments when they acknowledge, value, and integrate the views of the students about a topic into educational practices (Klemenčič, 2021). It should be noted that student views result from various conditions such as misconceptions, cultural backgrounds, and knowledge systems. In the case of evolutionary theory, the literature suggests that students’ initial views about this are a crucial element to consider in order to promote students’ understanding of this theory (Grossman & Fleet, 2017, Kampourakis, 2014; Kampourakis & Nehm, 2014; Larrain et al., 2021; Lucero et al., 2017).

Recent studies have focused on exploring the views of students about evolution in countries where there is little information documented in this respect, such as Chile (Larrain et al., 2021; Marín et al., 2021), Colombia (Archila & Molina, 2020; Marín et al., 2021), Brazil (Demetrio et al., 2021), and Greece (Mantelas & Mavrikaki, 2020). The present study seeks to contribute to this exploration, particularly in Colombia. The purpose of our exploration of students’ views is twofold. First, it aims to document the misconceptions about evolution among STEM/non-STEM majors. Second, it aims to determine whether their alternative conceptions of evolution are influenced by demographic variables. Specifically, we addressed the following research questions:

1. What are Colombian undergraduates’ misconceptions of evolution?
2. How are Colombian undergraduate students’ misconceptions of evolution related to demographic variables, namely, age, gender, education major, and semester of data collection?

## Literature Review

### Views About Evolution: an Area of Research in Science Education

In this article, the Darwinian theory of evolution—the most accepted theory of evolution—is defined as a “theory of descent with modification through natural selection” (Darwin, 1859, p. 343). It may be obvious to note that the study of students’ views about evolution started various decades ago and it is today a consolidated area of research in science education (Dudycha, 1934). Barnes et al. (2021) and Branch et al. (2021) report that even in the USA—probably the country where more studies have been conducted—there are still under-documented issues. It is thus not surprising to find in other parts of the globe statements of concern, such as “there are very few studies in the science education literature that have documented how Indonesians view evolution” (Aini et al., 2020, p. 714), “information about the knowledge and acceptance of evolution in Spain is scarce” (Gefaell et al., 2020, p. 3), “little is known about the views of university students in Colombia” (Archila & Molina, 2020, p. 1625), and “relatively little information is available regarding the level of acceptance of evolution and knowledge about evolution in different educational settings in Europe” (, p. 1).

In response to these gaps in the literature, recent studies have contributed to the improvement of our knowledge of the views of students, teachers, and the general public about evolution in different regions (not only in the USA). To give an example, in Flanders (Belgium) and the Netherlands, Pinxten et al. (2020) evidenced that the relative frequency of alternative conceptions elicited by the multiple-choice instrument “Conceptual Inventory of Natural Selection” was very similar in Flemish and Dutch in university freshmen (Biomedical and Veterinary sciences). Furthermore, these researchers found that “intention/need related to speciation” was the most common alternative conception in both samples. In a study conducted with Indonesian pre-service biology teachers, Rachmatullah et al. (2018) reported that about half of the sample (*N* = 22) revealed invalid teleological reasoning as they used the need/goal concept to explain evolutionary change. Also in Indonesia, Putri et al. (2017) examined the (1) concepts’ mastery and (2) misconceptions about evolution from biology-major university students. Their outcomes indicate that participants’ low understanding of evolution seems to be the cause of many of their misconceptions.

As part of a comparative study, 963 university students and 659 people who reported having at least a college-level education were surveyed in China and the USA, respectively (Zhang et al., 2022). Regarding participants’ knowledge about evolutionary theory, the results reveal that the mean evolution knowledge score (maximum possible score = 26) was not significantly (*p* = 0.206) different when comparing the Chinese sample (15.05 ± 3.37) and the US sample (15.29 ± 4.54). In another survey study carried out in Germany, the knowledge of evolution among students was examined (Kuschmierz et al., 2020a, 2020b). Participants (*N* = 406) included 136 biology undergraduate students (58.8% females and 40.4% males, 17–31 years old), 124 non-biology undergraduate students (55.6% females and 41.9% males, 18–37 years old), and 146 high school students (58.9% females and 41.1% males, 15–19 years old). The results show that, on a scale score from 0 to 29, biology undergraduates (*M*_score_ = 17.9) were more knowledgeable about evolution while high school students (*M*_score_ = 14.7) were less. With a mean score of 16.2, non-biology undergraduate students were found to be located between these two groups.

Larrain et al. (2021) explored the ideas about evolution among 137 Chilean students (62 females and 75 males, 11–12 years old). The outcomes reveal that participants showed a low knowledge of the theory of evolution. In Colombia, Archila and Molina (2020) report on 7 years of data generated by a three-question survey to explore views about evolution and creationism. Respondents were 1132 undergraduate students (16–23 years old) from 13 different majors (e.g., Anthropology, Biology, Civil engineering, Industrial engineering). This study is believed to be the first of this kind in Colombia. These academics arrived at two valuable conclusions: namely (1) a high percentage of the participating students held evolutionary views and (2) “students’ majors and age did not influence their views” (p. 1632). Gefaell et al. (2020) surveyed 981 third-year undergraduate students from the academic programs: Biology, Chemistry, English, History, and English Philology, at ten Spanish universities. These researchers found that participants showed a moderate (5.4 out of 10) knowledge of evolution although there were differences across different degree programs (Biology (6.5) > Chemistry (5.2) > History (4.8) > English Philology (4.4)).

In Brazil, Medina Tavares and Bobrowski (2018) examined the understanding of the theory of evolution by twenty-three undergraduate biology students in the process of pedagogical training. The results indicate that participants had a very low level of knowledge of evolutionary theory. For instance, 43.5% of the undergraduates considered that the statement, “Organisms tend to over-reproduce themselves” (p. 452), was not a part of Darwin’s theory of natural selection. The point of concern here is that this was actually one of the crucial notions of this scientist for developing such a theory. Besides, many participants resorted to the use of invalid teleological thinking to answer questions related to adaptation or natural selection. Even more discouraging is the fact that although participants were almost finishing their degrees, the majority of them acknowledged that they did not feel prepared to teach the theory of evolution to primary and secondary school students.

“What are the misconceptions of Science Teachers in the topic about Evolution?” (Abenes & Caballes, 2020, p. 33). This was one of the two questions that guided a survey study carried out with 54 science teachers in the Philippines. In general, twenty misconceptions were identified. In particular, some participants held misconceptions such as the idea that dinosaurs lived at the same time as the earliest human beings. The second question addressed in this study was related to the perception of participants about the sources of their misconceptions. Electronic media (37%) and textbooks (33.4%) were the top 2 most mentioned sources. Sanders and Makotsa (2016) also investigated the South African Senior Phase (Grade 7 to 9) curriculum statement and textbooks for the natural sciences as possible sources of unscientific ideas about evolution. To be specific, these scholars analyzed the Revised National Curriculum Statement on Natural Sciences and six Natural Sciences textbooks. They found scientifically incorrect statements in every document analyzed. An example is the overgeneralized and hence incorrect idea that fossils are preserved in stone. The problem here is that this ignores the existence of fossils found in ice and amber. Moreover, these authors detected unscientific notions such as the belief that individuals evolve by purposely initiating and controlling change in order to survive. Furthermore, fragmentation problems and poor sequencing were identified. Interestingly, in the textbooks, many of the statements identified revealed problematic use of language (e.g., anthropomorphic and teleological wording) in ways which implied or could lead to misconceptions.

Taking a course on evolution appears to contribute to dealing with misconceptions about evolution. Karataş (2020), for instance, examined and compared the misconceptions of Turkish participants (*N* = 190) who completed an evolution course (*n* = 91) and those who did not (*n* = 99). A key outcome was that the percentage of participants who evidenced misconception (43.4%) was higher in the group of students who did not take the evolution course than in their counterparts (15.3%). Some of the misconceptions revealed by the study include the following: (1) the association of evolution only with Darwin; (2) the assumption that evolution implies the effort to bring individuals to perfection; (3) the thought that apes were the ancestors of human beings (this misconception has been also reported in Indonesia (Aini et al., 2020; Ramadani & Ibana, 2020)); (4) the idea that an individual changes when conditions change in order to adapt; and (5) the link between evolution and metamorphosis. Another interesting finding was that 14 out of the 91 participating students held misconceptions, even though they had completed the course. Of course, the claim that biology instruction can be useful to address student misconceptions has also shown promising results in the USA (e.g., Doudna, 2016; Hawley & Sinatra, 2019; Sinatra and Danielson 2016).

### Multiple Instruments Available to Examine Views About Evolution

Zhang et al. (2022) observe that there is not one unique tool for examining the understanding of evolution. The great majority of studies developed to investigate individuals’ views about evolution tend to use surveys (questionnaires) as tools to collect data. The use of semi-structured interviews is not actually common. And studies that use both surveys and semi-structured interviews are scarce (e.g., Alanazi, 2021). There are also questionnaires that provide information about eventual relationships between views of evolution and religion (e.g., Marín et al., 2021; Silva et al. 2021). Likewise, there exists a battery of multiple questionnaires to measure the knowledge concerning evolution (see Kuschmierz et al., 2020a, 2020b, for a fuller discussion). In addition, it is possible to select items from other surveys so as to construct a new instrument adapted for specific purposes. To give an example, Archila and Molina (2020) constructed a three-question survey with items “that had been validated previously in the USA and had a history of use in the evolution education literature in their current form” (p. 1626). It is important to note that a questionnaire can serve educational practices in many ways. For instance, Buckberry and Burke da Silva (2012) administered a questionnaire before and after completing an introductory biology course in order to evaluate the effectiveness of this course in challenging misconceptions and increasing the acceptance of evolution.

Having stressed that there exist multiple questionnaires to examine views about evolution, it is worth mentioning that caution needs to be taken regarding the generalizability of contextual findings. According to Barnes et al. (2019), the choice of instrument can influence the results and conclusions of a study. Additionally, they provide evidence for the claim that the extent to which variables (e.g., evolution understanding) predict the results depends on the instrument used. With this in mind, these scholars invite us “to be critical of existing instruments and not [to rely on] an instrument’s popularity alone in making a decision about which instrument to use” (pp. 15–16). They also insist that the research questions should be recognized as key criteria when selecting any instrument.

## Significance of the Study

Students’ misconceptions about evolution, which are the focus of this study, have a notable place in the agenda of the research on evolution education. Nevertheless, most research has been conducted in the USA. Hence, several authors reiterate that it is of paramount importance to bridge this gap, carrying out studies in those countries where little (or no) information has been reported, since having a global understanding of this issue is a condition *sine qua non* for the development of effective, pragmatic, and realistic practices in evolution education (Aberilla et al., 2021; Marín et al., 2021). The present study is significant in terms of the country and the educational level researched. To be clear, our study was carried out in Colombia—a country, among many, in which research on university students’ misconceptions about evolution has not received substantial attention so far. More important, it expands the literature on evolution education by providing evidence from an under-researched Latin American country.

## Materials and Methods

### Sample and Sampling

The study took place over 5 years (ten academic semesters: from spring 2017 to spring 2022) in the same introductory biology course at a mid-sized private university in Bogotá, Colombia. This university has a high academic ranking in Latin America. This course was chosen for three reasons. First, the course has been developed to receive all undergraduates that want to enroll in it independently of their major. It is therefore common to find STEM and non-STEM majors in this course. Second, there is no age limit to enrollment. Thus, this course gave us the opportunity to access students from a wide age range. And third, special background in the theory of evolution is not required to enroll on this course. It is important to note that the last author is the usual instructor of this course.

The study was evaluated by and received approval from the university’s ethics committee (project code 9,520,001). According to Colombian law, data was used for research purposes only, confidentiality was duly addressed, and participants were given the opportunity to abandon the study whenever they wanted. Participants were undergraduates enrolled in the introductory biology course. Participation was completely voluntary, and participants were not offered any compensation. No personally identifiable information (e.g., name, Student ID) was asked for, to ensure the anonymous participation of each student. The instructor made clear to the students what the purpose of the study was. A total of 700 students completed the questionnaire. Nevertheless, only 547 questionnaires were considered after excluding those with missing information. Participants included undergraduates from STEM and non-STEM majors. The age range was 16 to 24 years (*M*_age_ = 18.7, *SD*_age_ = 1.75). Table 1 shows the characteristics of the participants. Age, gender, and education major are the demographic variables considered in this study since these can be explored in almost every university population (Aberilla et al., 2021; Kuschmierz et al., 2020a, 2020b; Ramadani & Ibana, 2020). Also, we included the semester in which each student completed the questionnaire as a variable to be examined.

**Table 1:** Number of participants by age, gender, education major, and semester group (*N* = 547)

Variable	Group	*n* (% = [*n*/547] × 100)
Age (years)	16–17	142 (25.9)
	18–19	248 (45.3)
	20–21	105 (19.1)
	22–24	52 (9.5)
Gender		
	Females	278 (50.8)
	Males	269 (49.1)
Education majors		
	STEM	436 (79.7)
	Non-STEM	111 (20.2)
Semester		
	Spring 2017–Fall 2017	96
	Spring 2018	64
	Spring 2019– Fall 2019	97
	Spring 2020– Fall 2020	104
	Spring 2021– Fall 2021	140
	Spring 2022	46

### Data Collection Instrument

In this cross-sectional study, we used the questionnaire developed by Buckberry and Burke da Silva (2012). The reason for this is that these scholars originally tested the questionnaire with undergraduate students (our target population). It should be pointed out that they also created this instrument to collect useful information about undergraduates’ acceptance and misconceptions of evolution rather than to obtain a “total score.” In this sense, we clarify that the estimation of a “total score” is not within the scope of our study. The questionnaire is composed of one “random”/ “accidents” statement (Question 8 in Buckberry & Burke da Silva, 2012, p. 269) and twenty-one “true”/ “false” statements regarding specific questions surrounding misconceptions relating to evolution and the acceptance of evolutionary theory. For example, “evolution is something that happens to individual organisms” (Item 7 in Buckberry & Burke da Silva, 2012, p. 269) is a false statement, while “the formation of complex structures, like the eye, can readily be explained by evolution” (Item 20 in Buckberry & Burke da Silva, 2012, p. 269) is a true one. In this article, we only included the eleven “true”/ “false” statements about misconceptions (all of which are false). To validate the “true”/ “false” nature of each item, two full professors with long experience in biological sciences individually coded each item as “true” or “false.” These experts are also co-authors of this article. Cohen’s kappa coefficient (Cohen, 1960) calculated was 0.90.

### Data Collection

All the data for the present cross-sectional study were obtained through the anonymous questionnaire (Buckberry & Burke da Silva, 2012). This is a rational data collection method because this instrument allows data to be collected to answer our research questions (Barnes et al., 2019). The entire questionnaire was presented to the students in Spanish, and, thus, they were tested in their first language. It is important to clarify that the instrument was translated from English to Spanish by a Spanish–English-German trilingual Full Professor at the Department of Biological Sciences (the last author). Translation was revised by an expert in Spanish–English bilingual science education (the first author) to remain as faithful as possible to the original meanings and wording. The questionnaire was administered by the last author in ten different academic semesters: 2017 (spring and fall semesters), 2018 (spring semester), 2019 (spring and fall semesters), 2020 (spring and fall semesters), 2021 (spring and fall semesters), and 2022 (spring semester). The administration of the instrument occurred on the first week of the introductory biology course to reduce instructor influence as far as possible. Survey respondents were asked to answer the questions, taking the time that was necessary to do so. Participants were informed that their answers would have no influence on their final grade and were given the opportunity to opt out of the study by not completing the questionnaire.

### Data Analysis

Data were analyzed using the Statistical Package for the Social Sciences (SPSS®) version 27. Analysis was carried out at two levels: (1) analysis of participants’ responses to the questionnaire and (2) analysis of possible relationships between students’ responses and demographic variables. In response to our first research question, undergraduates’ responses were analyzed in a descriptive way, using arithmetic mean, frequencies, percentages, and standard deviations (SD).

To address our second research question, a Kolmogorov–Smirnov test—a test of whether a distribution is normal—was conducted, with the result that the Sig. (*p*) values for misconceptions questions was < 0.000. This means that the assumption of normality was not met. Accordingly, nonparametric (non-grouped) statistical tests were applied. This entailed a cross-sectional analysis of differences in students’ responses across demographic variables to be carried out with the Mann–Whitney test (Mann & Whitney, 1947) for the variables “gender” and “education major” and with the Kruskal–Wallis test (Kruskal & Wallis, 1952) for the variables “age” and “semester,” as for these variables, there were more than two different categories (George & Mallery, 2022, p. 245). We decided to use the Mann–Whitney test and the Kruskal–Wallis test for two reasons. First, these tests are applicable to a range of different data sets since these do not require that the data sets to be compared are the same size. The second reason is that assumption of distribution is not required to apply these tests. A *p* < 0.05 was considered as statistically significant. It is important to clarify that, as recommended by Field (2018), we excluded from this cross-sectional analysis those questions in which Levene’s test—a test of homogeneity of variances—revealed that variances of students’ responses for each of the demographic variables differed significantly (*p* < 0.05). In other words, questions that did not meet the assumption of homogeneity were excluded.

### Methodological Limitations

Caution is advised regarding the interpretation and generalizability of our findings given that there are several methodological limitations in this study. One clear limitation is the relatively small sample size (*N* = 547) as well as the fact that respondents are drawn from the same university. Another noticeable weakness is that although data were collected from 2017 to 2022, we adopted a cross-sectional design (Clark et al., 2021, p. 50); our data were collected from a questionnaire that each participant completed just once. Thus, our study is limited to provide a mere “snapshot” of the 547 respondents’ misconceptions about evolution, although this weakness applies to nearly every cross-sectional study interested in examining these aspects concerning evolution education (e.g. Aberilla et al., 2021; Barnes et al., 2021; Branch et al., 2021). Also, despite the fact that the Mann–Whitney test and the Kruskal–Wallis test are legitimate nonparametric statistical tests (George & Mallery, 2022), their results should always be interpreted with caution. This largely explains why we excluded questions that did not meet the assumption of homogeneity, in order to reduce the risk of Type I error (false positive) (Field, 2018).

## Results

In this study, eleven questions were used to identify misconceptions about evolution. Table 2 presents the students’ responses to these questions. In this table, a higher mean indicates a lower level of misconception about each item. Our results reveal that nine out of the eleven items had media values above 0.70. The importance of these outcomes is that they provide evidence to claim that, in general, participants had a moderate understanding of evolution. In particular, we identified two items that deserve special attention. First, 58.5% of the students responded “false” (correct response) to the statement “evolution only occurs over millions of years” (Q5). This means that the rest (41.5%) of the undergraduates responded “true,” supporting this misconception. And second, very few of the students (5.4%) responded “false” to the statement “evolution involves individuals changing in order to adapt to their environment” (Q10). Thus, this finding suggests that the great majority (517/547) of the participants relied on this invalid teleological reasoning about evolution. Arguably, this misconception can become an obstacle for the students to understand one core element of evolutionary thought, namely “that organisms and the environment form dynamic systems that change continuously” (Noguera‑Solano et al., 2021, p. 910).

**Table 2:** Means, standard deviations, quantity, and percentages of correct responses per question about misconceptions (*N* = 547)

Item		Mean (SD)	Correct response
			*n* (% = [*n*/547] × 100)
1	Evolution is the same as “Natural Selection.”	0.83 (0.37)	457 (83.5)
2	Evolution is primarily concerned with the origin of humans	0.84 (0.36)	460 (84)
3	Evolution is something that happened only in the past; it is not happening now	0.94 (0.22)	517 (94.5)
4	Evolution is something that happens to individual organisms	0.80 (0.39)	443 (80.9)
5	Evolution only occurs over millions of years	0.58 (0.49)	320 (58.5)
6	There is actually very little evidence for evolution	0.91 (0.28)	499 (91.2)
7	One indication that evolution has not occurred is the total absence of “transition organisms” (those with traits intermediate between two different groups)	0.81 (0.38)	446 (81.5)
8	Fossils provide many problems which evolution cannot explain	0.74 (0.43)	405 (74)
9	Dinosaurs lived during the time of the early humans	0.95 (0.20)	522 (95.4)
10	Evolution involves individuals changing in order to adapt to their environment	0.05 (0.22)	30 (5.4)
11	There is actually considerable observable evidence against evolution	0.84 (0.36)	462 (84.4)

Table 3 shows the Mann–Whitney test outcomes for the variables “gender” (Q2, Q3, Q7, and Q9) and “education major” (Q1, Q3, Q4, Q6, Q7, and Q8). We found that males held lower levels of misconception about evolution in two (Q2 and Q9) out of the four questions analyzed. Nevertheless, the Mann–Whitney test results reveal that the students’ responses to these questions were not significantly different between males and females. Likewise, the results of this nonparametric test indicate that for the six questions analyzed, there were no significant differences between STEM students and non-STEM students. In particular, we found that STEM majors had lower levels of misconception about Q6, Q7, and Q8 than non-STEM majors, but these differences were not reliable since they were not statistically significant.

**Table 3:** Misconceptions about evolution: the Mann–Whitney test of participants’ responses (*N* = 547)

Variable	*n*	Q1	Q2	Q3	Q4
		Mean (SD) Mdn	Mean (SD) Mdn	Mean (SD) Mdn	Mean (SD) Mdn
Gender					
Females	278	-	0.83 (0.36) 1.0	0.94 (0.21) 1.0	-
Males	269	-	0.84 (0.36) 1.0	0.94 (0.23) 1.0	-
*U*		-	37,176.50	37,050.0	-
*Z*		-	–0.18	–0.46	-
*p-*value		-	0.85	0.64	-
Education major					
STEM	436	0.83 (0.37) 1.0	-	0.94 (0.22) 1.0	0.80 (0.39) 1.0
Non-STEM	111	0.83 (0.37) 1.0	-	0.94 (0.22) 1.0	0.82 (0.37) 1.0
*U*		24,126.0	-	24,174.0	23,622.50
*Z*		–0.07	-	–0.04	–0.57
*p-*value		0.94	-	0.96	0.56
Variable	*n*	Q6	Q7	Q8	Q9
		Mean (SD) Mdn	Mean (SD) Mdn	Mean (SD) Mdn	Mean (SD) Mdn
Gender					
Females	278	-	0.81 (0.38) 1.0	-	0.94 (0.18) 1.0
Males	269	-	0.81 (0.36) 1.0	-	0.96 (0.36) 1.0
*U*		-	37,208.0	-	36,763.50
*Z*		-	–0.14	-	–0.93
*p-value*		-	0.88	-	0.34
Education major					
STEM	436	0.91 (0.28) 1.0	0.82 (0.38) 1.0	0.74 (0.43) 1.0	-
Non-STEM	111	0.90 (0.28) 1.0	0.78 (0.41) 1.0	0.72 (0.44) 1.0	-
*U*		24,127.0	23,239.50	23,874.0	-
*Z*		–0.09	–0.95	–0.28	-
*p-value*		0.92	0.33	0.77	-

*Mdn* median; only questions that met the assumption of homogeneity (Levene’s test) were considered in the analysis

Table 4 presents the Kruskal–Wallis test findings. At first glance, there are differences in how age groups responded to Q1, Q5, Q7, and Q9. Nonetheless, we should stress that the Kruskal–Wallis test results reveal that these were not statistically significant. This means that undergraduates’ responses to these questions do not depend on their age. In the case of the variable “semester,” Q2 and Q6 were analyzed. We found that the Spring 2020–Fall 2020 semester group had higher levels of misconception about these two questions, but the Kruskal–Wallis test indicates that these outcomes were not statistically significant.

**Table 4:** Misconceptions about evolution: the Kruskal–Wallis test of participants’ responses (*N* = 547)

Variable	*n*	Q1	Q2	Q5	Q6	Q7	Q9
		Mean (SD) Mdn	Mean (SD) Mdn	Mean (SD) Mdn	Mean (SD) Mdn	Mean (SD) Mdn	Mean (SD) Mdn
Age groups							
16–17	142	0.85 (0.35) 1.0	-	0.57 (0.49) 1.0	-	0.81 (0.38) 1.0	0.95 (0.21) 1.0
18–19	248	0.83 (0.37) 1.0	-	0.58 (0.49) 1.0	-	0.81 (0.38) 1.0	0.95 (0.21) 1.0
20–21	105	0.84 (0.36) 1.0	-	0.59 (0.49) 1.0	-	0.80 (0.39) 1.0	0.95 (0.21) 1.0
22–24	52	0.78 (0.41) 1.0	-	0.63 (0.48) 1.0	-	0.80 (0.39) 1.0	0.98 (0.13) 1.0
*H*(3)		1.27	-	0.68	-	0.06	0.92
*p-*value		0.73	-	0.87	-	0.99	0.81
Semester groups							
S 2017–F 2017	96	-	0.84 (0.34) 1.0	-	0.92 (0.26) 1.0	-	-
S 2018	64	-	0.87 (0.33) 1.0	-	0.90 (0.29) 1.0	-	-
S 2019–F 2019	97	-	0.84 (0.36) 1.0	-	0.90 (0.29) 1.0	-	-
S 2020–F 2020	104	-	0.80 (0.39) 1.0	-	0.88 (0.32) 1.0	-	-
S 2021–F 2021	140	-	0.85 (0.35) 1.0	-	0.92 (0.27) 1.0	-	-
S 2022	46	-	0.82 (0.38) 1.0	-	0.93 (0.24) 1.0	-	-
*H*(5)		-	1.59	-	1.75	-	-
*p-*value		-	0.90	-	0.88	-	-

*S* spring, *F* fall; only questions that met the assumption of homogeneity (Levene’s test) were considered in the analysis

## Discussion

A recent bibliometric mapping analysis of trends in science education research revealed that the examination of students’ acceptance and misconceptions of evolution is an area of great interest for science education stakeholders (Wang et al., 2022). Despite this interest, very little is known in this respect in countries such as Chile (Larrain et al., 2021; Marín et al., 2021), Colombia (Archila & Molina, 2020; Marín et al., 2021), Brazil (Demetrio et al., 2021), and Greece (Mantelas & Mavrikaki, 2020). Thus, in this article, we report the misconceptions about evolution among STEM/non-STEM majors of different ages, genders, and semester of administration of the 21-item questionnaire. Additionally, we have documented whether demographic variables influence the students’ responses. The importance of this cross-sectional study is that it was carried out in a Latin American country, providing baseline information for the development of a global understanding of the role of evolution education in the world. Therefore, we reiterate that to the best of our knowledge, this is the first study investigating STEM/non-STEM majors’ misconceptions about evolution in Colombia. In the following paragraphs, the results are discussed with respect to the two research questions and context, but also in relation to the literature on evolution education.

First Research Question—“What are Colombian undergraduates’ misconceptions about evolution?” An overview of the results (Table 2) shows that students had a moderate understanding of evolution. These outcomes extend emergent literature as they provide a more nuanced understanding of our knowledge about evolution education among university students in Colombia (Archila & Molina, 2020). Furthermore, these are consistent with the idea that “it is quite reasonable to expect students to enter into classroom with a number of misconceptions about evolutionary theory” (Deniz & Borgerding, 2018b, p. 4). Unfortunately, as Reydon (2021) observes, instructors seldom show genuine interest in knowing about the misconceptions hold by their students. Also, instructors rarely use students’ misconceptions about evolution as a platform to promote meaningful learning. He calls for evolution education practices that recognize students’ misconceptions as useful educational resources for engaging students in authentic learning experiences, rather than obstacles requiring complete replacement.

Additionally, we found that 95.4% of the students responded “false” to the statement “dinosaurs lived during the time of early humans,” this being the best responded statement. In the study conducted by Buckberry and Burke da Silva (2012) with undergraduates in Australia, 74.4% of the participating students responded “false” to this statement (Q9, in Buckberry & Burke da Silva, 2012, appears as Q16, p. 269). It is noteworthy that several academics (e.g., Alters & Nelson, 2002) have reported that few people choose “false” when responding to this statement, whether they are university students (e.g., Haldane et al., 2022) or the general public (e.g., Relethford, 2017). It is even more interesting to realize that Haldane et al. (2022) as well as Relethford (2017) report from probably the most documented country: the USA. Here, we are reporting from Colombia, one of the countries where the fewest studies have been conducted. Relethford (2017) maintains that some cartoons (e.g., the classic American cartoon, The Flintstones®) and movies (e.g., the American science fiction action film, Jurassic Park®) show situations of modern human beings and dinosaurs living at the same time. This is a sound explanation of why “a substantial percentage of Americans” (p. 33) agree with the misconception that dinosaurs lived at the same time as the earliest human beings. Furthermore, he insists that this is a misconception that concerns times where there exists an accurate history of life on earth, supported by modern geoscience techniques that allow scientists to evaluate data about the dates of fossils. It is thus clear that the widespread claim that many people commonly subscribe to this misconception should be interpreted with caution as our outcomes do not support this. Arguably, this contradictory result involving samples from Australia, the USA, and Colombia highlights the need for science education stakeholders to hear and value the voices of people from as many different countries as possible when talking about evolution education in the world.

Another interesting point of discussion is that our outcomes reinforce Buckberry and Burke da Silva’s (2012) claim that the most critical student misconception relates to the statement “evolution involves individuals changing in order to adapt to their environment” (Q10, in Buckberry & Burke da Silva, 2012, appears as Q17, p. 269). Although these authors maintain that instructors can use students’ responses to Q10 as one indicator of possible “Lamarckian and/or teleological ideas of evolution” (p. 271) held by their students, it is important to clarify that, as Kampourakis and Zogza (2007) argue, in some cases, it could be inaccurate to interpret students’ preconceptions as “Lamarckian” because these are essentially different from the original ideas of Jean‑Baptiste Lamarck. In similar fashion, Noguera‑Solano et al. (2021) invite us to use Lamarck’s evolutionary framework as a springboard for promoting students’ awareness of the vital role of argument, debate, and critique in scientific practice. No doubt, this invitation is coherent with respect to the claim that history and the philosophy of science can be used as educational resources to engage students in productive argumentation practices (e.g., argumentative interaction) (Archila, 2015; Archila et al., 2020a). Nevertheless, this is unlikely to occur if instructors do not appreciate the true educational power and potential of the evolutionary ideas that Lamarck developed.

Moreover, Reydon (2021) emphasizes that it is unproductive simply to label as a wrong conception the students’ misconception that individuals can change to better fit into their environments (Q10 in Table 2). Accordingly, he reiterates that one possibility (among many) for instructors to address this misconception is through the organization of a whole-class discussion that revolves around key features such as the concepts of fitness and environment in biology. In this way, students would be provided with the opportunity to become aware of the scientific value contained in this misconception, even though taken literally, it is inaccurate. An important question that should be considered is whether we can claim that this misconception indicates that our participating students possess a teleological idea of evolution. Kampourakis (2020) contends that characterizing students’ misconceptions as “teleological” can be misleading. He explains that one reason for this is that the problem with many of these misconceptions is not teleology per se but the underlying “design stance.” Discussing this in more detail, this researcher maintains that the claim that a feature may exist in order to perform a function is not necessarily unsound, because if a feature has been selected for the function that it performs; clearly this function is the reason that it exists, and thus, this is a solid teleological explanation. In reality, the problem is the claim that this function can be attributed to a design teleology—assumes that (i) the intentions of an external agent (e.g., God, nature) or (ii) the needs of the organism itself explain why a feature exists. In short, given that the statement “evolution involves individuals changing in order to adapt to their environment” shows a needs-based design teleology, we can claim that the students (94.5%) who responded “true” to this statement used invalid teleological reasoning about evolution. Thus, this outcome supports Kampourakis’s (2020) standpoint that “it is the design stance and not teleology that we need to address in evolution education” (p. 6). Probably, putting this standpoint into practice would contribute to dealing with the issue reported by Buckberry and Burke da Silva (2012) To be precise, they observed that undergraduates rarely change the idea that organisms can change to better fit into their environments, even if they were asked this question both at the beginning and at the end of a 13-week evolutionary course.

In addition, we found a limited understanding of microevolution among participants (Q5 in Table 2). This is a matter of concern, given the importance of microevolution for human health and disease. Fouad (2018), for instance, argues that the understanding of microevolutionary changes is a pillar of many of the practical applications of evolution, such as developing flu vaccines or preventing antibiotic resistance in human pathogens. Our result clearly adds evidence to her call to “help students understand the distinction between microevolution and macroevolution, rather than simply using the more ambiguous term “evolution” as a catch-all” (p. 32).

Second Research Question—“How are Colombian undergraduate students’ misconceptions of evolution related to demographic variables, namely, age, gender, education major, and semester of data collection?” Results from the Mann–Whitney and Kruskal–Wallis tests reveal that the students’ responses to the questions analyzed about misconceptions of evolution were not significantly related to any of the four independent variables examined in the current study (Tables 3 and 4). Arguably, a key contribution of this cross-sectional study is to the scarcity of research on evolution education in Colombia dealing with the relationships between alternative student conceptions and demographic variables. Specifically, evidence from this study suggests that despite apparent relationships, these were not reliable since the relationships were not statistically significant. In this sense, these outcomes are in line with Abenes and Caballes’s (2020) reflection that not all factors predict student misconceptions of biological evolution, and those that do, do not work in the same manner. This consideration gives us an idea about the complexity involved in this issue.

Furthermore, it is interesting to note that the semester in which the questionnaire was administered did not influence students’ responses. This is a key result that offers insights about the period between Spring 2017 and Spring 2022, indicating that the moderate level of understanding of evolution seems not to have significantly changed in 5 years in Colombia. The reason is straightforward: the promotion of a genuine understanding of evolution is not a priority in this country. Peñaloza et al. (2021) highlight relevant characteristics of the Colombian curriculum context that we consider useful to better interpret this result. First, in this country, evolution is taught at the 8th or 9th grade levels of secondary education. Typical students in these grades are 13–14 years old. It is therefore obvious that despite having been an increase in the last few decades in the number of calls in various countries to implement evolution in early science education (e.g., Adler et al., 2022; Campos & Sá-Pinto, 2013; Chanet & Lusignan, 2009; Frejd et al., 2022; Nadelson et al., 2009; Sá-Pinto et al., 2021; Vázquez-Ben & Bugallo-Rodríguez, 2018; Wagler, 2012), in Colombia, teaching evolution in early childhood and primary school level is not yet the norm. In this regard, our results corroborate that the need for education on evolution from the initial years of schooling is warranted. Another characteristic to consider is that Colombian state schools must guarantee the freedom to teach evolution. Thus, in these schools, teachers cannot be censored for religious reasons. One consequence of this is that evolution education could vary from state schools to private schools. Naturally, the situation becomes more complicated because, as Peñaloza et al. (2021) document, in Colombia, the evolution teaching practices of biology teachers seem to be influenced by their views on relationships between science and religion.

## Implications

“Evolution is the most profound and powerful idea to have been conceived in the last two centuries” (Diamond, 2001, p. vii). However, evolution education faces numerous challenges, such as instructors’ lack of sufficient knowledge of evolution (Siani et al., 2022), instructors’ poor pedagogical content knowledge to diagnose and deal with student misconceptions of biological evolution (Hartelt et al., 2022), student opposition to learning evolution (Siani et al., 2022), and the absence of research on misconceptions about evolution in countries, such as Chile, Colombia, Brazil, and Greece. Deniz and Borgerding (2018b) stress that the documentation of evolution education issues around the globe is a necessary step to overcome the multiple challenges of preparing citizens to think critically about the theory of evolution. In this regard, the findings from this study offer several key implications for evolution education practice.

First of all, our results highlight the need to recognize the diagnosis of student misconceptions as a substantial step forward in the promotion of a more sophisticated understanding of the theory of evolution among STEM/non-STEM students. A point to bear in mind here is that simply diagnosing student misconceptions is not likely to lead to genuine learning about evolution. Much of the reason for this is that learning implies “constructing meaning and reflecting critically on this meaning” (Archila et al., 2022, p. 3). Therefore, instructors need to be prepared to see student misconceptions as valuable resources for providing students with explicit opportunities to construct meaning about evolutionary theory and to become engaged in critical reflection about this meaning. As Smith and Siegel (2019) point out, criticality is especially desirable to avoid indoctrination—accepting, believing, or embracing something without reason—in evolution education. In short, instructors should assume student misconceptions as a valuable foundation upon which to build a critical understanding of the theory of evolution, which, of course, respects the social and cultural context of each country. In Colombia, for example, there are traditions and beliefs held by indigenous communities that should not be overlooked (Archila et al., 2021).

The results seem to further imply that evolution education stakeholders in Colombia should not only encourage the distinction between micro and macroevolution within the design of new curriculum materials, but also support instructors on how to effectively translate the curriculum into practice. From a pedagogical standpoint, such a distinction is relevant to provide students with concrete opportunities to develop a full understanding of evolution. By the same token, the finding that STEM/non-STEM majors’ misconceptions about evolution were not significantly different should be understood as a valuable opportunity to use the distinction between micro and macroevolution as a platform to enrich interdisciplinary skills (e.g., creativity and collaborative work). Archila et al. (2020b), for instance, have demonstrated that these skills can be fostered among both STEM students and non-STEM students when specific evolution concepts are discussed in the light of the nature of science rather than in an isolated fashion. Clearly, this implies instructor training and support as “it cannot be assumed that biology teachers with extensive backgrounds in biology have enough knowledge of evolution, natural selection, or NOS [nature of science]” (Siani et al., 2022, p. 766).
